# Geochemical baseline establishment and accumulation characteristics of soil heavy metals in Sabaochaqu watershed at the source of Yangtze River, Qinghai-Tibet Plateau

**DOI:** 10.1038/s41598-024-62628-5

**Published:** 2024-09-20

**Authors:** Jiufen Liu, Cang Gong, Changhai Tan, Lang Wen, Ziqi Li, Xiaohuang Liu, Zhongfang Yang

**Affiliations:** 1 China University of Geosciences, Beijing, China; 2 National Research Center for Geoanalysis (Key Laboratory of Eco-Geochemistry, Ministry of Natural Resources), Beijing, China; 3 Research Center of Applied Geology of China Geological Survey, Chengdu, China; 4 Key Laboratory of Natural Resource Coupling Process and Effects, Beijing, 100055 China; 5 Natural Resources Comprehensive Survey Command Center of China Geological Survey, Beijing, China; 6 Technology Innovation Center for Analysis and Detection of the Elemental Speciation and Emerging Contaminants, China Geological Survey, Kunming, 650111 China

**Keywords:** Qinghai-Tibet Plateau, Tuotuo river, Soil heavy metal, Geochemical baseline, Background value, Biogeochemistry, Environmental sciences

## Abstract

The establishment of soil geochemical baseline and heavy metal pollution assessment in the Qinghai-Tibet Plateau is of great significance for guiding environmental management in the high-cold and high-altitude regions. A total of 126 topsoil samples (0–20 cm) were collected and the contents of Cu, Pb, Zn, Ni, Cr, Cd, As and Hg were determined in the Sabaochaqu basin of the Tuotuo River, the source of the Yangtze River, in the Tibetan Plateau. The baseline values of 8 heavy metals were determined by mathematical statistics, iterative 2times standard deviation method, cumulative frequency and reference element standardization, and the soil heavy metal pollution in the study area was assessed by enrichment factor method and pollution index method. The results showed that the average contents of As, Cd, Cr, Cu, Hg, Ni, Pb and Zn were 31.84, 0.29, 66.07, 17.35, 0.021, 27.86, 49.35 and 88.56 mg/kg, respectively. Baseline values were 22.24, 0.217, 64.16, 15.69, 0.0191, 26.46, 34.91, and 68.62 mg/kg, respectively. There is a great difference between the baseline value of soil heavy metals in study area and the Xizang soil background value, especially the baseline value of Cd was 2.68 times of its background value. The results of the pollution evaluation based on the baseline values showed that the 8 heavy metals were slightly enriched, and the overall pollution status was light pollution, and measures should be taken to control and manage them. The research results can provide a reference value for the evaluation of soil heavy metal pollution in the source region of the Yangtze River, and also provide a theoretical basis for the construction of soil heavy metal baseline values in similar high-cold and high-altitude regions.

## Introduction

After years of urbanization and industrialization, soil heavy metal (HM) pollution has become a serious environmental problem in the world^1–3^. It changes the physical and chemical properties of soil, reduces soil quality, soil and crops, and further endangers human health through the food chain^4,5^. Soil HM pollution was more prominent in some areas of China. According to the national soil pollution survey, the over-standard rates of soil Cd, Hg, As, Cu, Pb, Cr, Zn and Ni were 7.0%, 1.6%, 2.7%, 2.1%, 1.5%, 1.1%, 0.9% and 4.8%, respectively^6^. On January 11, 2024, the “Opinions of the Central Committee of the Communist Party of China and the State Council on Comprehensively Promoting the Construction of a Beautiful China” emphasized the need to carry out soil pollution source prevention and control actions, and promote the traceability and full coverage of HM pollution in agricultural land soil in stages.

A large number of research results have shown that the pollution assessment^7–10^, source analysis^11–14^ and risk assessment^15–18^ of HMs in soil need to be realized by geochemical baseline value or soil background value. However, the previous soil HM pollution assessment and risk assessment were mostly based on the national soil environmental quality agricultural land soil pollution Risk control standard (trial) (GB15618-2018), soil environmental quality construction land soil pollution risk control standard (trial) (GB36600-2018), China soil element background value, provincial and regional background value, phase comparison values of neighboring regions or historical data are used as reference standards for evaluation. However, China has a vast territory and diverse soil types, and the natural abundance of HMs in the native soil environment in different regions is different. Therefore, it is not scientific to use unified standards to evaluate or assess soil HM pollution and accumulation degree and risk, which may lead to inaccurate evaluation results^19–21^. As Guan et al.^22^ found in their study of agricultural soils in Wuwei, Gansu Province, using the background values of the soil in the Hexi Corridor where the research area is located as the standard, Ti, V, Cr, Mn, Cu, and Zn were only 0.39–0.71 times the background values, all at a pollution-free level, while Pb was 3.61 times the background value, indicating severe pollution. However, using a geochemical baseline as the standard, the average content of 8 HMs was higher than the baseline value, which was 1.004–1.27 times the baseline value, indicating pollution accumulation. Source analysis shows that the main sources of pollution were atmospheric deposition, agricultural activities, and industrial activities, supporting the rationality of using a geochemical baseline as the accumulation evaluation standard. Other scholars also found that the regional background values of some HMs were significantly different from the baseline values^23,24^. Without considering the influence of natural factors such as the parent material and the heterogeneity of the soil forming process on the natural abundance of HMs in soil^25^, it is difficult to distinguish whether the accumulation of HMs in soil belongs to the primary high geological background or the input of unreasonable human activities. Using a unified background value as the standard for evaluation may reduce or amplify the pollution level. Furthermore, the phenomenon of “under-protection” or “over-protection” appears when guiding pollution control and treatment^21,26^. Therefore, more and more attention has been paid to soil baseline values.

The geochemical baseline refers to the natural variation of the concentration of chemical elements in the earth's surface, which is defined as the upper limit of the geochemical background (undisturbed by human activities) or the lower limit affected by human activities^24,27^. The geochemical baseline was first proposed by the Global Geochemical Baseline (IGCP360) project and the International Geological Correlation Program (IGCP259)^27,28^, and is an important reference for distinguishing between natural and anthropogenic environmental impacts^29–31^. Known as the “roof of the world” or the “third pole”, the Qinghai-Tibet Plateau is one of the regions least affected by human activities at present, and the content of HMs in the ecosystem of the Qinghai-Tibet Plateau under natural conditions is relatively low. In recent years, with the gradual acceleration of climate change, the exploitation and utilization of natural resources, and the development of secondary and tertiary industries, the ecological environment of the Qinghai-Tibet Plateau has been gradually affected, and its soil system has suffered from HM pollution to a certain extent^32^. As the main basin of the headwater of the Yangtze River in the hinterland of the Qinghai-Tibet Plateau, the variation characteristics of soil HMs in the Tuotuo River basin have attracted the attention of domestic scholars, and a few of them have carried out soil HM accumulation assessment^33,34^. However, due to the lack of suitable reference standards, the soil background values of Qinghai Province or Tibet were usually selected as the reference standards, resulting in insufficient accuracy of the assessment results. Therefore, on the basis of investigating the HM content in soil in the Sabaozhaqu watershed of Tuotuo river, the source of the Yangtze river, this study compared and determined the soil HM geochemical baselines by using mathematical statistics, iterative 2times standard deviation method, cumulative frequency and reference element standardization, and used the baseline values to evaluate the accumulation characteristics of HMs in soil. The research results can make up for the shortcomings of soil HM baseline research in the Tuotuo river basin at the source of the Yangtze river, provide important theoretical references for the construction of soil HM baseline values in high-cold and high-altitude areas, and also provide feasible reference basis for soil pollution evaluation in the source area of the Yangtze river. The aim was to provide guidance for the implementation of soil pollution source prevention and control actions in the “Opinions of the Central Committee of the Communist Party of China and the State Council on Comprehensively Promoting the Construction of a Beautiful China”, provide scientific basis for promoting the tracing and remediation of HM pollution in soil, as well as the protection and management of major rivers such as the Yangtze and Yellow Rivers.

## Materials and methods

### Study area

The research area is located in the hinterland of the Qinghai-Tibet Plateau, and is located in the northernmost part of Amdo County, Tibet Autonomous Region, north of Hoh Xili Reserve, Zhiduo County, Qinghai Province, east of Yanshiping Town, Amdo County (90°32°47.62″–91°49°13.06″E, 33°23°16.46″-34°41°31.47″N). The climate is an alpine grassland ecosystem with cold and arid, thin air, strong wind and open terrain. Affected by the cold air activity near the surface and the strong west wind from the air, the wind speed is high, and the annual average number of strong wind days exceeds 110 days. The temperature and pressure are low, the temperature difference between day and night is large, and the radiation is strong, and the freezing period is from September to April of each year. The average annual air pressure is 584.3 mb and the average annual temperature is − 4.2 °C. The climate in the basin is dry and cold, with less precipitation and poor natural environment. More than 90% of the area is uninhabited.

### Sampling and analysis

**Figure 1:**
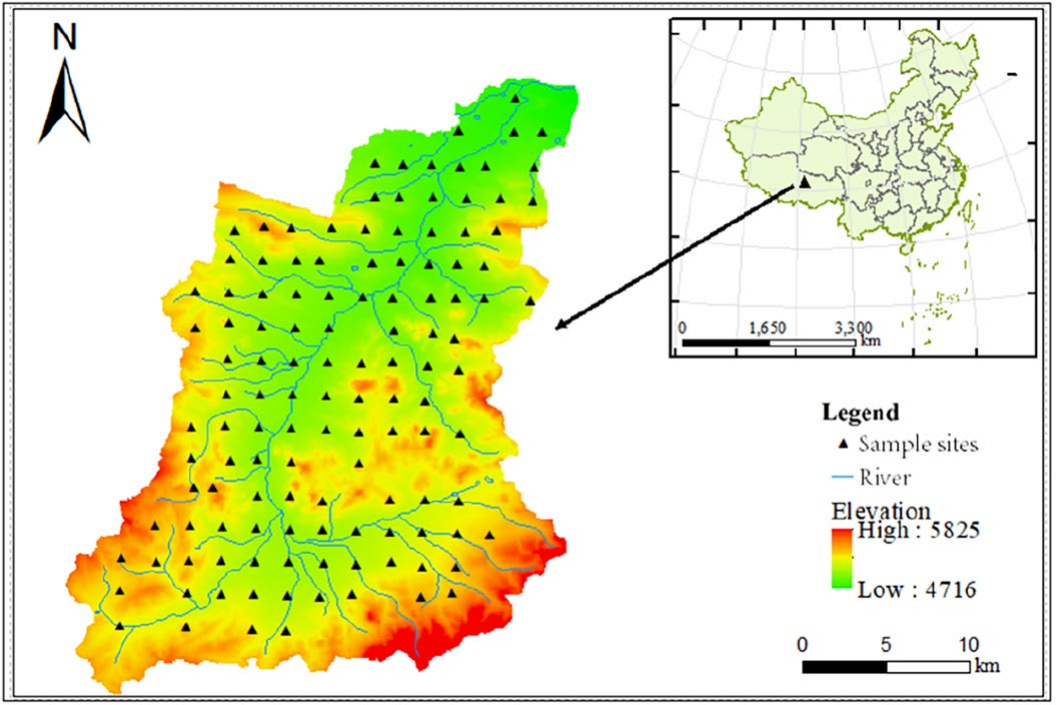
The location of the study area and sampling sites (Map were generated with software ArcMap10.8 http://www.esri.com/).

The field sampling was completed in 2022, and a total of 126 pieces of topsoil samples (0–20 cm) were collected with reference to the specification for geochemical evaluation of 1:250,000 land quality, and the sampling location was shown in Fig. 1. In order to improve the representativeness of the soil samples, the sampling points were evenly distributed in a 4 km^2^ sampling grid, and the distance between each sampling point was required to be more than 2 km. 3–5 multi-points were collected within a range of 100 m around the sampling point to form a sample, and the original weight of the combined samples was more than 1 kg. Portable GPS was used to locate the sampling points. Visible impurities were removed from all collected samples and air-dried at room temperature.

### Test analysis and data processing

The analytical tests were completed by Chengdu Integrated Rock and Mineral Testing Center of Sichuan Geological and Mineral Exploration and Development Bureau. The analytical quality was strictly carried out in accordance with the Specification for Geochemical Evaluation of Land Quality (DZ/T0295-2016). Cu, Pb, Zn, Ni, Cr, Cd, Sc, La, Mn and V were determined by X-ray fluorescence and inductively coupled plasma optical/mass spectrometry, and As, Hg and Sbwere determined by atomic fluorescence method. The quality of analytical tests was controlled by means of insertion of eight soil standard substances at national level, repeatability test, anomaly check and blank test. The quality parameters of the test meet the requirements of the specification, and the result data are real and credible. The detection limits of each index analytical test are shown in Table 1.

**Table 1:** Detection limit of analyzed indicators.

Parameter	Detection limit (μg/g)	Parameter	Detection limit (μg/g)
As	0.2	Zn	0.03
Cd	0.01	Sc	0.1
Cr	5	La	0.2
Cu	0.5	Mn	0.02
Hg	0.003	V	0.3
Ni	0.2	Sb	0.2
Pb	0.7		

### Geochemical baseline calculation methodology

#### Mathematical statistics method

Soil HM content was tested for distribution and parameters such as arithmetic mean, geometric mean and median value were counted. Baseline values were expressed as arithmetic means if they obeyed a normal distribution, geometric means if they obeyed a lognormal distribution, and median values for elements that obeyed neither a normal nor a lognormal distribution^35^.

#### Iterative 2times standard deviation method

The mean (${\overline{\text{X}}}$) and standard deviation (σ) of the initial dataset were first calculated, and values outside the range of ${\overline{\text{X}}}$ ± 2σ were eliminated. This step was repeated for the resulting new dataset until all remaining values were within ${\overline{\text{X}}}$ ± 2σ and the remaining dataset conformed to a normal or lognormal distribution, using the arithmetic mean or geometric mean of the final retained data as the geochemical baseline values^23,24^.

#### Cumulative frequency method

The cumulative frequency method calculates the geochemical baseline of soil HMs while distinguishing the effects of anthropogenic activities^24^. The method involves fitting a relative cumulative frequency curve using relative cumulative frequency as the Y-axis and elemental content as the X-axis. The fitted curve will have 0, 1, or 2 inflection points representing natural, anthropogenic, and anomalous concentration boundaries, respectively. To determine the inflection point, this study used the method of determining the inflection point by Fan et al.^36^, which starts from the bottom of the curve and calculates the determination coefficient R^2^ of the fitting curve between each point after the starting point and the starting point in sequence. Initially, there will be a series of fluctuations in R^2^, and then it will gradually rise to the highest point, which is the 1st inflection point; then, using the 1st inflection point as a new starting point, the fitted curve R^2^ is calculated between the points after the 1st inflection point and the 1st inflection point in turn until the 2nd inflection point is found. If the inflection point is 0, all sample data represent the geochemical baseline range of the HM; if the inflection point is 1, the data below the inflection point represent the geochemical baseline range of the HM element, while the data above the inflection point represent the portion that has been affected by human activities; if the inflection point is 2, then comparing the similarity of the pattern of the frequency distributions between the two inflection points to the patterns of the frequency distributions prior to the first inflection point and those after the second inflection point, respectively. If it is close to the first inflection point, the first inflection point is chosen as the upper limit for baseline value calculation, and vice versa for the second inflection point^23,36,37^.

#### Reference element standardization method

The basic idea of reference element standardization method is to select the inert elements in the geochemical process as the standard, and use the correlation between the active elements and the inert elements to judge the enrichment of active elements^27^. This means selecting inert elements that meet certain conditions as reference, normalizing soil HM elements, removing outliers until all sample points fall within the 95% confidence interval, and statistically analyzing the subset of data formed to obtain a geochemical baseline^23^. The selected inert elements are abundant in the earth's crust, are highly resistant to weathering, reflect soil evolutionary processes, are mainly derived from natural matrices, have mass fractions that are sensitive to anthropogenic inputs, are not easily affected by a variety of natural effects (elements that are strongly influenced by mining should be excluded), have a clear correlation with the active elements, and the amount of the soil deposit can be accurately determined^23,27,36,38^. In this study, the geochemical baseline was determined by analyzing the correlations between a variety of inert elements (e.g., Sb, Ti, Sc, V, and La) and reactive elements, selecting the optimal set of correlations, and establishing a regression linear equation using points within the 95% confidence limits of the sample data^27^.

### Cumulative characteristics evaluation method

#### Enrichment factor method

Enrichment factor (EF) is an important indicator for distinguishing between natural and anthropogenic sources of HMs in soil and is calculated as.

1 $$ {\text{EF }} = \, \left[ {{\text{M}}_{i} /{\text{M}}_{ie} } \right]_{{\text{S}}} / \, \left[ {{\text{M}}_{i} /{\text{ M}}_{ie} } \right]_{{\text{B}}} $$

where [M_*i*_/M_*ie*_]_S_ is the ratio of the content of HM *i* to the reference element in the soil sample, and [M_*i*_/ M_*ie*_]_B_ is the ratio of the content of soil HM *i* to the background or baseline value of the reference element. Trace elements Sc, V, Ti, etc., which have no obvious anthropogenic origin in the soil, are often chosen as reference elements^39^. In this study, the correlation analysis and the results of the reference element standardization method (Sect. 3.2.4) were combined, and As was calculated with Sb as the reference element, Cd with Mn as the reference element, Cr, Cu and Zn with Sc as the reference element, Hg and Ni with V as the reference element, and Pb with La as the reference element. Enrichment factors are usually categorized into 6 classes: no enrichment (EF ≤ 1), slightly enriched (1 < EF ≤ 2), moderately enriched (2 < EF ≤ 5), significantly enriched (5 < EF ≤ 20), strongly enriched (20 < EF ≤ 40), and very strongly enriched (EF > 40).

#### Pollution index method

The pollution index method contains single-factor pollution index (PI) and comprehensive pollution index (SPI), PI is based on the full amount of individual soil HMs, which can simply and effectively assess the degree of pollution caused by individual soil HMs; SPI can effectively reflect the comprehensive pollution degree of different HMs to the soil environment, which are calculated by the formulas as follows, respectively:

2 $$ {\text{PI}} = \frac{{C_{i} }}{{S_{i} }} $$

3 $$ {\text{SPI}} = \sqrt {\frac{{\left( {\frac{{C_{i} }}{{S_{i} }}} \right)_{max} + \left( {\frac{{C_{i} }}{{S_{i} }}} \right)_{ave} }}{2}} $$

where PI denotes the single-factor pollution index of HM* i*; C_*i*_ denotes the measured value of soil HM *i*; S_*i*_ denotes the background or baseline value of HM* i*; and SPI denotes the composite pollution index of all HMs at a sampling point. Five pollution levels were classified according to PI and SPI: clean (safe) (< 0.7), alert level (0.7–1.0), lightly polluted (1.0–2.0), moderately polluted (2.0–3.0) and heavily polluted (≥ 3.0).

### Data statistics and analysis

ArcGIS10.8 was used to draw the sampling map, SPSS26.0 was used to analyze the data with descriptive statistics and correlation analysis, and Origin2019 was used to complete the plotting.

## Results and discussion

### Distribution characteristics of soil HMs in study area

The test results and descriptive statistics of 126 surface soil samples from the study area were shown in Table 2. The arithmetic mean contents of As, Cd, Cr, Cu, Hg, Ni, Pb, and Zn were 31.84, 0.29, 66.07, 17.35, 0.021, 27.86, 49.35 and 88.56 mg/kg, respectively. The Kolmogorov–Smirnov normality test showed that Cr and Ni were normally distributed, and Pb had a lognormal normal distribution, and As, Cd, Cu, Hg and Zn were skewed. Except for Cr and Ni, the coefficients of variation were more than 30%, among which As, Cd, Pb and Zn were more than 50%, which showed that there was obvious spatial heterogeneity of soil HMs, implying that there were some geographical differences and some anthropogenic perturbations in the content of these HMs in the soils of the study area.

**Table 2:** Test results and statistical data for HMs in the study area.

Item	As	Cd	Cr	Cu	Hg	Ni	Pb	Zn
Max (mg/kg)	157	2.18	110	87.2	0.066	48.4	584	582
Min (mg/kg)	10.2	0.083	41.0	8.71	0.010	12.1	17.9	38.3
Arithmetic mean (mg/kg)	31.84	0.295	66.07	17.35	0.021	27.86	49.35	88.56
Geometric mean (mg/kg)	27.38	0.252	65.01	16.42	0.020	26.83	41.52	79.51
Median (mg/kg)	25.10	0.251	66.15	16.50	0.020	27.10	40.65	75.65
Standard deviation (mg/kg)	23.21	0.24	11.97	7.85	0.007	7.58	52.60	58.35
Coefficient of variation (%)	72.9	80.7	18.1	45.3	33.8	27.2	106.6	65.9
Skewness	3.34	4.92	0.44	6.01	2.42	0.44	8.58	5.36
kurtosis	13.49	33.15	0.52	50.82	11.01	-0.03	86.64	41.01
Distribution characteristics	Skew distribution	Skew distribution	Normal distribution	Skew distribution	Skew distribution	Normal distribution	Lognormal distribution	Skew distribution

### Determination of soil HM baseline values

#### Mathematical statistics method

According to the statistical and testing results (Table 2), Cr and Ni are normally distributed, and their baseline values are represented by the arithmetic mean value; Pb is lognormal and represented by the geometric mean value; As, Cd, Cu, Hg and Zn are skewed and represented by the median value. Therefore, the baseline values of As, Cd, Cr, Cu, Hg, Ni, Pb and Zn based on mathematical statistics were 25.10, 0.252, 66.07, 16.5, 0.020, 27.86, 41.52 and 75.65 mg/kg, respectively.

#### Iterative 2times standard deviation method

The mean ± 2σ was used to continuously remove the outliers in the initial data set of soil HM content, and the Kolmogorov–Smirnov test was performed on the data subset formed after iteration. The results showed that As (9 iterations), Cr (3 iterations) and Cu (6 iterations) were normally distributed, and the baseline values were represented by the arithmetic mean. Cd (10 iterations), Hg (6 iterations), Ni (3 iterations), Pb (9 iterations), and Zn (9 iterations) follow a lognormal distribution, with a geometric mean representing the baseline value. Therefore, the baseline values of As, Cd, Cr, Cu, Hg, Ni, Pb and Zn by the iterative 2times standard deviation method were 22.42, 0.204, 65.06, 15.85, 0.0184, 26.39, 34.90 and 68.60 mg/kg, respectively.

#### Cumulative frequency method

**Figure 2:**
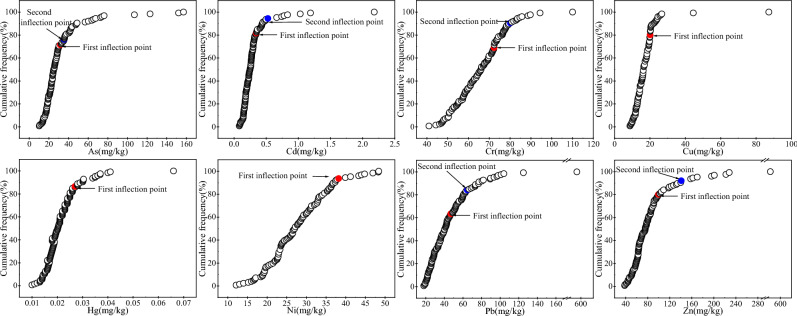
Cumulative frequency curves for HMs in the study soils and arrows indicate the positions of inflexion.

The cumulative frequency distribution of HMs in soil in the study area is shown in Fig. 2. The results show that there is only 1 inflection point for Cu, Hg and Ni, and 2 inflection points for other HMs. A small part of the data falls outside the first or second inflection point of the curve, which means that this part of the sample was affected by humans to a certain extent, but the cumulative frequency of this part of the sample is mostly above 80%, which shows that its proportion was very low. The baseline values of As, Cd, Cr, Cu, Hg, Ni, Pb and Zn based on cumulative frequency method were 22.04, 0.222, 63.73, 15.25, 0.0193, 26.72, 31.93 and 69.62 mg/kg, respectively.

#### Reference element standardization method

**Figure 3:**
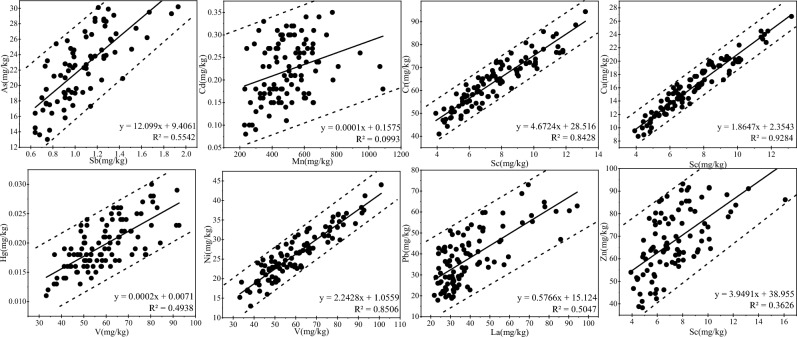
Relationship curves between eight HMs with inert elements.

Combined with previous studies, Sc, La, Mn, Sb and V all meet the conditions of standardized reference elements^25,27,36^. Table 3 shows the Pearson correlation analysis results between five reference elements and HM elements in soil. It can be seen that each HM responds to different inert elements with the most significant correlation. As and Sb, Cr, Cu and Zn and Sc, Hg and Ni and V were all significantly positively correlated. It is worth noting that although the correlation between Pb and La and Cd and Mn was lower than 0.3, the correlation between Pb and Cd with other inert elements were worse. In the case that there was no alternative inert element, La and Mn were the best choices for this study. In this study, reference elements with the highest correlation coefficient with each HM were selected, and the data within the confidence limit of 95% were used for regression analysis (Fig. 3). A linear regression equation was established, and the mean value of the corresponding reference element content was substituted into the regression equation, that is, the geochemical baseline value of soil HM elements was obtained. The baseline values of As, Cd, Cr, Cu, Hg, Ni, Pb and Zn based on reference element standardization method were 22.25, 0.225, 63.68, 15.96, 0.0196, 26.28, 37.89 and 67.64 mg/kg, respectively.

**Table 3:** Correlation between inert elements with HMs in topsoil.

	As	Cd	Cr	Cu	Hg	Ni	Pb	Zn
Sc	0.135	0.232**	0.812**	0.598**	0.455**	0.677**	–0.019	0.325**
La	0.023	–0.115	-0.193*	–0.108	–0.170	0.340**	0.190*	0.013
Mn	0.189*	0.245**	0.513**	0.367**	0.360**	0.543**	0.088	0.327**
Sb	0.572**	0.218*	0.399**	0.219*	0.301**	0.239**	0.095	0.295**
V	0.143	0.220*	0.787**	0.589**	0.465**	0.698**	–0.035	0.301**

After the standardization of reference elements, except that the correlation coefficient between Cd and Mn of 0.0993 (Fig. 3) was significantly lower than that before standardization of 0.245 (Table 3), the correlation coefficient between other HMs and reference elements after standardization remained basically the same or increased with that before standardization. This may be because the sample points of other HMs affected by human activities were less and removed in the standardization process, while the sample points of Cd affected by human activities were more and cannot be effectively removed during the standardization of reference elements.

#### Comparison of baseline values determined by different methods

Table 4 shows the geochemical baseline values of soil HMs in the study area determined by the mathematical statistics method, the iterative 2-times standard deviation method, the cumulative frequency method, and the reference element standardization method, respectively. It was obvious that the baseline values determined by the mathematical statistics method are significantly higher than those determined by the other three methods, which was mainly due to the fact that the high values were not eliminated when the mathematical statistics method was used. Similar results were obtained in study of Wu et al.^24^.

**Table 4:** Geochemical baseline values (mg/kg) as determined by different methods.

Item	Baseline values						Background values		
	Mathematical statistics	Iterative 2-times standard deviation	Cumulative frequency	Reference element standardization	Study area	Lhasa^35^	Hoh Xil^42^	Tibet^41^	Qinghai Province^41^
As	25.10	22.42	22.04	22.25	22.24	20	13. 28	19.7	14.0
Cd	0.252	0.204	0.222	0.225	0.217	0.13	0. 11	0.081	0.137
Cr	66.07	65.06	63.73	63.68	64.16	42	47. 8	76.6	70.1
Cu	16.50	15.85	15.25	15.96	15.69	23	22. 3	21.9	22.2
Hg	0.020	0.0184	0.0193	0.0196	0.0191	0.079	/	0.024	0.020
Ni	27.86	26.39	26.72	26.28	26.46	21	23. 59	32.1	29.6
Pb	41.52	34.90	31.93	37.89	34.91	31	16. 54	29.1	20.9
Zn	75.65	68.60	69.62	67.64	68.62	70	51. 41	74	80.3

Comparatively, the iterative 2-times standard deviation method, the cumulative frequency method, and the reference element standardization method were suitable for the construction of elemental baseline values where regional anthropogenic contamination was light. The iterative method removes the effect of outliers more completely^40^. The cumulative frequency curve method belongs to the statistical method, which can not only obtain the geochemical baseline range of HMs, but also identify the anomalies caused by human activities from the natural anomalies^23,27,36^. In particular, the method of determining the inflection points by calculating the coefficient of determination of the regression curves R^2^ improved by Fan et al.^36^ can quickly and effectively determine the location and number of the inflection points, which solves the problems of the conventional cumulative frequency curve method, which can’t be easily derived directly from the curve to determine its baseline value and the identification of inflection points mainly relies on subjective judgment. A straight line, it was not easy to directly derive its baseline value from the curve, and the problem that the identification of inflection points mainly relies on subjective judgment. The standardized method is based on the premise that no anthropogenic interference has been introduced into the reference elements, and the inert elements in the geochemical process are used as reference standards to determine the enrichment of active elements by using their correlation with the active elements. This method was first used to study elemental concentrations in marine and estuarine sediments, and was later extended to HM geochemical baseline studies in soils^21,23,25,26,30,31^. In general, the baseline values of soil HMs in the study area determined using the iterative 2-times standard deviation method, cumulative frequency method and reference element standardization method were close to each other for As, Cd, Cu, Hg and Ni, except for some differences in Cr, Pb and Zn. It is worth mentioning that although the correlation between Cd and the reference element Mn was low in the reference element standardization method, the obtained geochemical baseline value of Cd of 0.225 mg/kg was similar to that of 0.222 mg/kg by the cumulative frequency method and 0.204 mg/kg by the iterative 2-times standard deviation method, suggesting that the selection of the reference element Mn was reasonable. For this reason, in this paper, the average of the baseline values determined using these three methods was used as the geochemical baseline values of soil HMs in the study area, namely, As 22.24, Cd 0.217, Cr 64.16, Cu 15.69, Hg 0.0191, Ni 26.46, Pb 34.91 and Zn 68.62 mg/kg.

Comparing the determined geochemical baseline values of the soils in the study area with the background values of the soils in Tibet^41^ (Table 3), the baseline values of As, Cd, and Pb were significantly higher than their background values, especially the baseline value of Cd, which was 2.68 times of its background value; while the baseline values of Cr, Cu, Hg, Ni, and Zn were significantly lower than their background values, which were only 0.72–0.93 times of their background values. Comparison of the geochemical baseline values in study area with the background values in the neighboring region of Qinghai Province^41^ also shows that the baseline values of As, Cd and Pb were significantly higher than their background values, and the baseline values of Cr, Cu, Hg, Ni and Zn were significantly lower than their background values. Compared with the soil background values of Hoh Xil in northern Tibet adjacent to the study area^42^, except that the baseline value of Cu was lower than the background value, the baseline values of As, Cd, Cr, Ni and Pb in the study area were higher than those in Lhasa City, the capital of Xizang, while the baseline values of Cu, Hg and Zn in the study area were lower than those in Lhasa City^35^. It can be seen that there were obvious differences between the baseline value of the research area and the large-scale background value, and there were also obvious differences between the baseline value and the background value of soil HMs in adjacent or similar areas, which further reflects the shortcomings of the large-scale background value in the analysis and application of specific research areas. Similar differences have also been reported in existing studies^23,30,31,38,43^. This difference may be caused by the regional variability of the Tuo Tuo river Sabao Chachu basin and the temporal specificity of the survey and analysis, whereby the metal elements in the surface environment at different times and in the region are affected by environmental factors and undergo a complex process of migration and transformation, resulting in temporal and spatial differences in baseline values. In addition, the environmental background value refers to the original concentration of the chemical composition or elements of natural substances (including water bodies, soils, plants, atmosphere, etc. in the environment) when they were not or seldom affected by human activities^38,40^; while the environmental geochemical baseline was defined as the instantaneous natural concentration of the chemical elements or substances of the Earth's surface layer in a specific medium (soil, sediment, etc.) under specific environmental conditions (climate, time, organisms, matrices, anthropogenic effects, etc.)^25,38,44^. In contrast to the environmental background value, the environmental geochemical baseline not only reflects the level of content of elements or substances, but also the range of their temporal and spatial distribution, which to a certain extent can reflect the environmental perturbations brought about by human beings or (and) nature. With the long-term accumulation of human activities and the rapid development of modern industry and agriculture, it has become increasingly difficult to find soil environments that are completely unaffected by human activities^38^.

In addition, due to the limitations of the mismatch of time and space scales and other factors, the lack of environmental background values in soil environmental quality studies in some specific regions often results in the inability to obtain the desired reference benchmark values for HMs in the study area. Adopting large-scale environmental background values as the reference standard may distort the evaluation results. Therefore, the establishment of an environmental geochemical baseline around the study area is an important means of effectively compensating for the inaccuracy of the assessment results due to the lack of baseline values.

### Soil HMs accumulation characterization in study area

#### Soil HMs enrichment levels

**Figure 4:**
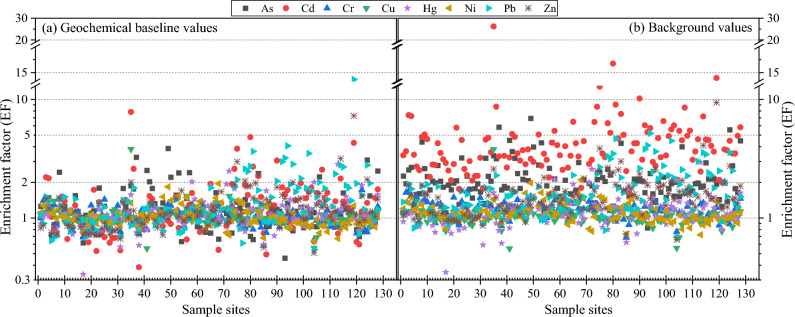
Scatter diagram of soil HMs enrichment factor (EF) based on geochemical baseline values (**a**) and background values (**b**) in this study.

The enrichment levels of soil HMs in the study area were evaluated using the defined geochemical baseline values and Tibet background values, respectively, and the results were shown in Fig. 4. By comparing the mean values of the enrichment factors of each HM element, it can be seen that the enrichment levels of the 8 HMs in the study area based on the baseline values (Fig. 4a) were in the order of Pb(1.47) > Cd(1.34) > Zn(1.23) > As(1.22) > Hg(1.12) > Cu(1.05) > Ni(1.04) > Cr(1.01), and the enrichment factors were higher than 1, which showed a slight enrichment (1 < EF ≤ 2) level in general. From the sampling points, the sample points at the level of no enrichment (EF ≤ 1) were Ni(52.38%) > Cr(46.83%) > Cu(41.27%) > As(39.68%) > Hg(38.89%) > Cd(38.1%) > Pb(38.1%) > Zn(37.3%); at the slightly enriched (1 < EF ≤ 2) level, the sample sites were Cu(57.94%) = Hg(57.94%) > Zn(57.14%) > Cr(53.17%) > Cd(51.59%) > As(50.79%) > Pb(47.62%) = Ni(47.62%); 13.49%, 9.52%, 9.52%, 4.76%, 3.17%, and 0.79% of the points of Pb, As, Cd, Zn, Hg, and Cu, respectively, were at the moderately enriched (2 < EF ≤ 5) level; and one point of Cd, Pb, and Zn, respectively, reached the significantly enriched (5 < EF ≤ 20) level. The overall accumulation levels of the 8 HMs based on the background values (Fig. 4b) were in the order of Cd(4.49) > As(2.20) > Pb(1.88) > Zn(1.59) > Cr(1.18) > Hg(1.16) > Ni(1.12) > Cu(1.05), with Cd and As as a whole reaching the medium enrichment level, and the rest of the HMs in general at the mild enrichment level. Cd, As, Pb, Zn, Hg, Cr, Cu, Ni each had 70.63%, 42.86%, 23.81%, 13.49%, 3.17%, 0.79%, 0.79%, 0.79% and 0.79% of the sample points were at the moderately enriched level; 24.60%, 2.38%, 1.59% and 0.79% of the sample points of Cd, As, Pb, and Zn respectively were at the significantly enriched level; in particular, one sample point of Cd was at the strongly enriched (20 < EF ≤ 40) level.

Comparison of the HM enrichment factors based on background values with the results based on baseline values showed that there were large differences in HMs except for Cu, Zn and Ni, which were not significantly different, and the evaluation results of Cd, As, Zn, Pb and Cr based on background values were 3.34, 1.80, 1.29, 1.28 and 1.17 times higher than the results of the evaluations using baseline values, respectively. It implies that the evaluation results using large scale environmental background values reflect a higher level of HMs enrichment in the study area, while the evaluation results using geochemical baseline reflect a relatively low level of HMs enrichment in the study area. This was mainly attributed to the difference between the geochemical baseline values of soil HMs in the study area and the Tibet soil background values, and many studies have reached similar conclusions^29,36,38,44,45^, which further validates that the use of the large-scale environmental background values as a reference standard may distort the evaluation results when a particular area was studied.

#### Soil HMs contamination levels

**Figure 5:**
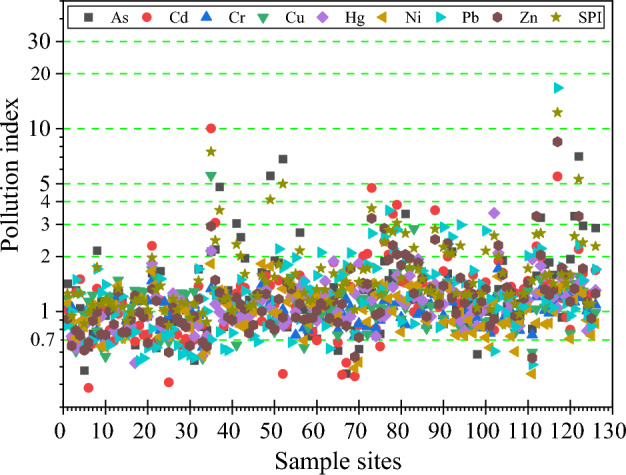
Scatter diagram of soil HMs enrichment factor (EF) based on geochemical baseline values in this study.

The evaluation of single factor pollution index (PI) and comprehensive pollution index (SPI) based on environmental geochemical baseline values as reference standards shows that (Fig. 5). The PI values of As, Cd, Cr, Cu, Hg, Ni, Pb and Zn in the study area were 0.46–7.06, 0.38–10.05, 0.64–1.71, 0.56–5.56, 0.52–3.46, 0.46–1.83, 0.51–16.73 and 0.56–8.48 respectively. The average PI values of 8 HMs were in descending order: As (1.43) > Pb (1.41) > Cd (1.36) > Zn (1.29) > Hg (1.12) > Cu (1.11) > Ni (1.05) > Cr (1.03), which means that all the 8 HMs in the study area were at the level of mild pollution. From the sampling points, the clean (PI < 0.7) levels were Ni(15.08%) > Pb(12.7%) > Zn(8.73%) > Cu(7.94%) = Cr(7.94%) = As(7.94%) > Hg(4.76%) > Cd(1.59%), and the alert level (0.7 ≤ PI < 1.0) level was Cd(42.86%) = Hg(42.86%) > Cu(37.3%) > Cr(34.13%) > Zn(33.33%) > Pb(26.98%) > As(25.4%) > Ni(23.02%), and at the lightly polluted (1.0 ≤ PI < 2.0) level was Zn(56.35%) > Cd(55.56%) > Cu(54.76%) > As(53.97%) > Hg(50%) > Pb(49.21%) > Ni(47.62%) = Cr(47.62%); at the level of medium contamination (2.0 ≤ PI < 3.0), the sample sites were in the order of Ni(12.7%) > Cr(7.14%) > As( 6.35%) > Pb(5.56%) > Hg(1.59%) > Zn(0.79%) > Cd(0.00%) = Cu(0.00%); As, Cd, Zn, Pb, Cu, and Hg had 8, 7, 4, 2, 1, and 1 sample sites at the heavy contamination (PI ≥ 3.0) level, respectively. It can be seen that the highest percentage of sample points for the 8 HMs were at mildly polluted levels, followed by those at alert level levels. The SPI of the 8 HMs in the study area ranged from 0.84 to 12.27 with a mean value of 1.75, showing overall mild pollution levels. Among them, 0.00%, 7.94%, 68.25%, 17.46% and 6.35% of the sample sites were in clean, alert level, lightly polluted, moderately polluted and heavily polluted levels, respectively. The Tibetan Plateau region is relatively underdeveloped, sparsely populated and environmentally clean in terms of industrial and agricultural development, but the neighboring regions such as South Asia and the Sichuan basin are characterized by strong human activities, especially in South Asian countries such as India and Bangladesh, which emit large quantities of HM pollutants and carbonaceous aerosols due to their continuously growing populations and rapid development of industry and agriculture^46,47^. In addition, HM pollutants were emitted into the atmosphere from the burning of cow dung and firewood in the daily lives of local residents, and from religious activities^48,49^. These HMs released into the atmosphere can be transported over long distances to the Tibetan Plateau, where they can affect the soil and other ecosystems through the “cold trap effect”^47,50,51^.

## Conclusions

Taking the Sabao Chaqu watershed of the Tuotuo River in the Yangtze River of the Qinghai-Tibet Plateau as the study area, the geochemical baselines of HMs Cd, Hg, As, Cu, Pb, Cr, Zn and Ni were determined by mathematical statistics, iterative 2-times standard deviation, cumulative frequency method and reference element standardization method, respectively, in the high-cold and high-altitude regions. The results showed that the baseline values determined by mathematical statistics were significantly higher than those determined by the other three methods, and the results determined by the other three methods were similar. The average value of the results of the following three methods was used as the geochemical baseline value of soil HMs in the study area. The baseline values of As, Cd and Pb were significantly higher than the background values of Tibet soil, while the baseline values of Cr, Cu, Hg, Ni and Zn were lower than the background values, which means that using the background values of Tibet soil as the reference standard for pollution assessment of this region will distort the evaluation results. Using baseline values as the reference standard, the results showed that the soil in the study area was generally slightly polluted with HMs, indicating that although the area was sparsely populated, it was also affected by human activities. The research results can provide reference for the related research of high-cold and high-altitude frozen areas.

The environmental geochemical baseline establishes the evaluation basis of the small scales area in space, which can make up for the lack of reference value or the fuzzy definition of the evaluation results caused by the use of large scales background value. In particular, the use of environmental geochemical baselines in alpine and high-altitude areas can be more accurate in assessment, and can effectively guide the formulation, management and implementation of environmental policies such as soil pollution prevention, tracking and remediation in alpine and high-altitude areas.

## Data Availability

All data generated or analyzed during this study are included in this published article.
